# Adherence to the Mediterranean Diet and Lifestyle Characteristics of University Students in Cyprus: A Cross-Sectional Survey

**DOI:** 10.1155/2016/2742841

**Published:** 2016-05-16

**Authors:** Elena Hadjimbei, George Botsaris, Vassilis Gekas, Andrie G. Panayiotou

**Affiliations:** ^1^Department of Agricultural Sciences, Biotechnology and Food Science, Cyprus University of Technology, 3036 Limassol, Cyprus; ^2^Cyprus International Institute for Environmental and Public Health in Association with the Harvard T. H. Chan School of Public Health, Cyprus University of Technology, 3041 Limassol, Cyprus

## Abstract

*Objective*. To assess dietary-related habits among young adults.* Design and Setting*. Dietary habits were assessed cross-sectionally, using a self-completed questionnaire in 193 students enrolled in public and private universities in Cyprus. Adherence to the Mediterranean diet was evaluated using the validated KIDMED index. BMI was estimated based on weight and height measurements.* Results*. The mean BMI was 23.31 (±3.98). The mean adherence score to the Mediterranean diet was 6.0 (IQR 4 to 8), with 26.9% of students being classified as high adherers and 21.8% as low adherers to the Mediterranean diet. About 32% of students consumed a second serving of fruit and vegetables more than once a day, whereas 26% reported going more than once a week to a fast-food restaurant and 31% consumed sweets and candy several times a day. On the other hand, 76% of participants reported consumption of at least two dairy products daily and 88% use olive oil at home. The majority consume coffee 2-3 times per day.* Conclusions*. Results support a shift from traditional healthy diets to more unhealthy eating patterns. However, we also report a high dairy intake and use of olive oil. Tailored-made strategies targeting the young adult population could be warranted.

## 1. Introduction

Young adulthood, defined as 18–25 years of age, is an important transitional period from adolescence to adulthood, during which long-term health behavior patterns are formed and established. This life stage is critical as many changes occur during it, such as the development of self-identity, leaving home, and increased autonomy in decision-making [1].

Young adults should have good dietary habits with adequate nutrient intakes, not only for overall good health, but also because the skeletal development continues during that period. Although approximately 90% of peak bone mass is attained by the age of 18 years, bone mass keeps growing until around age 30 [2]. However young adults tend to follow a poor diet, marked by low consumption of fruits and vegetables and high consumption of fast food and sugar-sweetened beverages [3]. This can influence not only their concurrent health but also their future risk for a number of chronic diseases at a later age. The Mediterranean diet pattern, originating from the traditional Mediterranean diet, has been consistently linked to a lower risk for a number of chronic diseases such as cardiovascular disease [4] and cancer [5].

Adherence to the Mediterranean pattern is therefore important in improving overall health, whereas young adulthood may be an important time for intervening and establishing long-term health behaviors. Given that a large proportion of young adults enroll in universities, university campuses represent a prime setting for data-driven health promotion intervention efforts.

Nonetheless, data on the diet of young adults, especially in Mediterranean countries such as Cyprus, are lacking. Therefore the aim of the study was to investigate the level of adherence to the traditional Mediterranean diet pattern overall and to specific dietary components, among Cypriot young adults identified through universities; thus providing much needed evidence for intervention programs targeting this often overlooked age group.

## 2. Methods

### 2.1. Study Participants

A total of 193 young adults aged 18–25 years participated in the present study from October to December 2014 through a convenience sampling. During this period a study researcher (dietitian, who provided help with the questions and who measured height and weight) visited two university campuses, one public (Cyprus University of Technology, Limassol) and one private (University of Central Lancashire in Cyprus, Larnaca) asking students to self-complete an anonymous questionnaire on baseline characteristics, including the following questions: “are you currently on a diet” (yes/no), “do you currently exercise” (yes/no), “do you currently take protein supplements” (yes/no), “do you currently smoke” (yes/no), and “how often do you drink coffee, tea, wine, beer, whiskey-vodka” (4–6 times/day; 2-3 times/day; 1 time/day; 1–4 times/week; 1–3 times/month; few times/year or never). All data were collected anonymously and completion of the questionnaire was considered informed consent.

Adherence to the Mediterranean diet was evaluated by the KIDMED index (Mediterranean Diet Quality Index for children and adolescents) [6] which includes 16 questions based on the principles of the Mediterranean diet, where those denoting a positive aspect with regard to the Mediterranean diet are assigned a value of +1 and those with a negative aspect −1. A total score ≤ 3 implies a very low diet quality, a score between 4 and 7 implies a diet that needs improvement to adjust intake to Mediterranean patterns, and a score ≥ 8 indicates optimal adherence to the Mediterranean diet.

Weight and height were also measured with the use of a portable digital scale and stadiometer at the standing position without shoes by the same study researcher. Body mass index (BMI) was calculated as weight/height^2^ (Kg/m^2^) and used in the assessment of overweight and obesity among young adults according to the International Obesity Task Force (IOTF) age- and sex-specific BMI cutoffs [7].

### 2.2. Statistical Methods

Continuous variables are presented as mean ± SD, whereas categorical variables are presented as frequencies. The normality of variables distribution was tested with the Kolmogorov-Smirnov test. The chi-square test was used to evaluate associations between the categorical variables and the Student's independent *t*-test and ANOVA methods were applied to evaluate differences in mean values of continuous variables as appropriate. A two-sided *p* value of less than 0.05 was considered statistically significant. Data were analyzed using SPSS v.20.0 (SPSS Inc., Chicago, IL) and Microsoft Office Excel 2007.

## 3. Results

### 3.1. Demographic Characteristics of the Participants

Out of 193 participants, 87 (45.1%) were men and 106 (54.9%) were women with a mean age of 20.56 (±1.85) years. The mean height was 169.06 (±9.53) cm and the mean weight was 67.09 (±14.9) Kg. The majority of participants came from the districts of Larnaca and Limassol, reflecting the location of the universities sampled. Overall characteristics of the subjects and for men and women separately are presented in Table 1.

### 3.2. Body Mass Index

**Figure 1:**
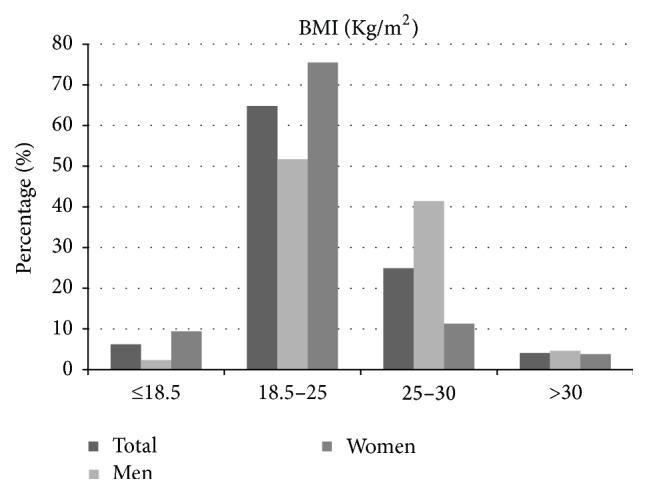
Distribution of BMI categories in all study participants and in men and women separately.

The mean body mass index (BMI) of study participants was 23.31 (±3.98) Kg/m^2^ with 6.2% of them being classified as underweight, 64.8% as normal weight, 24.9% as overweight, and 4.1% as obese (Figure 1). Differences between sexes were significant for all categories with more women being in the “underweight” category (9.4%) compared to men (2.3%) (*p* < 0.001) and more men in the “overweight” category (41.4%) compared to women (11.3%) (*p* < 0.001). BMI groups for all participants and by sex are shown in Table 1.

Participants who were on a diet at the time of the study had significantly higher mean BMI values (25.54 ± 5.18 Kg/m^2^) compared to those who were not (22.94 ± 3.62 Kg/m^2^) (*p* = 0.001).

When looking at other dietary variables that could be associated with an individual's BMI, such as breakfast consumption, fruit and vegetable consumption, fast-food consumption, smoking, exercise, and taking protein supplements, none of them was found to be significantly associated with mean BMI and results were similar for men and women, although more men were current smokers or exercised. Data are shown in Table 2.

### 3.3. Adherence to the Mediterranean Diet (KIDMED Score)

**Figure 2:**
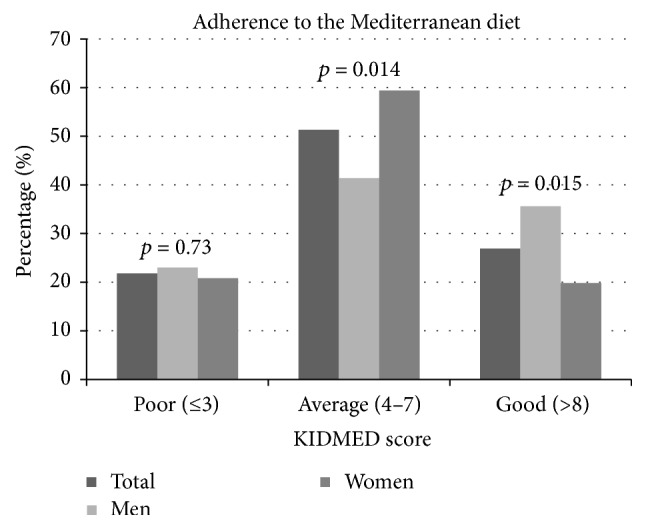
Distribution of KIDMED score categories in all and men and women separately.

The median KIDMED score in the study participants was 6.00 (IQR: 4 to 8), with men having a slightly but statistically nonsignificant higher score compared to women (5.95 versus 5.46, resp., *p* = 0.25). When looking at categories of adherence in all, about half (51.3%) were in the “intermediate adherence” to the Mediterranean diet category (total score between 4 and 7), 21.8% were in the “low adherence” category (total score of ≤3), and 26.9% were in the “high adherence” category (total score ≥ 8) (Figure 2). Again, there were differences between men and women, with more men in this age group being in the “high adherence” category compared to women (35.6% versus 19.8%, resp., *p* = 0.015). KIDMED score categories for all and men and women separately are shown graphically in Figure 2.

In addition to overall adherence to the Mediterranean diet pattern, specific dietary categories included in the KIDMED index and their possible differences between men and women were also studied. With regard to the main nutritional categories of the Mediterranean diet, 73.1% of all study participants consumed a fruit/fruit juice daily, 56.5% consumed fresh or cooked vegetables daily, and 47.7% consumed pulses >1 per week. Additionally, 76.2% ate two yogurts and/or 40 g cheese daily, with 88.1% consuming a dairy product for breakfast, whereas 87.6% use olive oil at home. As perhaps expected in university students, ~70.0% ate pasta or rice almost daily (≥5 times per week), ~40% skipped breakfast, and 26% ate at a fast-food restaurants >1 per week. Consumption of the abovementioned categories did not differ between men and women. Statistically significant differences in consumption between men and women were observed only in having a second serving of fruit daily (*p* = 0.020) and regular nut consumption (at least 2-3 per week) (*p* < 0.000) with higher values in men for both categories, whereas women tended to consume sweets more often (*p* = 0.04). Results are shown in detail in Table 3.

When looking at other personal characteristics that could be associated with adherence to the Mediterranean diet, not smoking (*p* = 0.005) and currently exercising (*p* < 0.001) were significantly associated with a higher adherence score (KIDMED score for not smoking = 6.01 ± 2.93 versus 4.61 ± 2.82 for smoking and 6.54 ± 2.87 versus 4.62 ± 2.70 for not exercising), indicating an overall healthier lifestyle. “Currently being on a diet” was not associated with adherence (5.96 ± 2.08 versus 5.64 ± 3.08; *p* = 0.59).

### 3.4. Beverage Consumption

The majority of participants (32.5%) consume coffee 2-3 times per day, with another 23% consuming coffee once per day. With regard to tea, about half of study participants (49.2%) drink tea 1–3 times per month or even more rarely. Regarding alcohol consumption, the majority of participants (38.7%) drink wine a few times per year with another 26.7% drinking wine 1–3 times per month and 27.2% drinking wine 1 to 4 times per week; the majority (37.2%) drinks rarely beer with another 27.7% drinking beer 1–3 times per month and 26.2% drinking beer 1 to 4 times per week; about half (50.3%) of all study participants drink whiskey or vodka a few times per year or never with another 25.1% drinking these beverages 1–3 times per month.

Statistically significant differences in beverage consumption between men and women were observed for beer (*p* = 0.000) and whiskey-vodka consumption (*p* = 0.012) with higher values in men for both categories (Table 4).

## 4. Discussion

Since the transition from adolescence to young adulthood is a critical period during which young people adopt and establish lasting health behavior patterns it is important to examine the dietary habits of this unique age group. In the present study we investigated dietary-related habits among Cypriot young adults.

The majority of our participants (64.8%) had a normal BMI. Our results are in line with a previous study conducted in Cyprus in children and adolescents where 62.8% of subjects were also shown to have normal weight [8]. Moreover Savva et al. [9] indicated that the prevalence of obesity (8.1%) and overweight (20.1%) in children and adolescents in Cyprus has increased substantially over a decade, mainly in rural areas and in school-aged boys. Our results showed that among young adults the prevalence of obesity was lower (4.1%) and the prevalence of overweight a little higher (24.9%).

The majority of young females were classified as normal weight (75.5%). It is concerning that about 9.4% of women were underweight. On the other hand, 41.4% of young males were classified as overweight. However, given that young men of that age exercise more than women, as also supported by our data (Table 1), BMI may not be a very good index for them because of the increased muscle mass [10] and therefore our results should be interpreted with caution. It is useful to note that 12.4% of the study sample was taking supplements at the time of the survey (Table 1) and of those 21.8% were males further supporting the above.

In the present study, only 26.9% of young adults had an optimal Mediterranean diet. Previous studies conducted on children and adolescents reported similar results to ours, namely, that there is a low to moderate adherence to the principles of the Mediterranean diet [11–13]. However, we did see a rather normal distribution among the groups, with about half in the average, middle category and the other half split in the two extremes. A key finding of the study was that although median KIDMED score did not differ significantly between men and women, young women were less likely to be in the “high adherers” category compared to men (19.8% versus 35.6% for women and men resp., *p* = 0.015). Previous studies had suggested that women have a better dietary profile than men [14]. However these studies were conducted on older populations and may not be directly comparable to our study population. When looking at individual index items, women in the study ate less fruit/vegetables and nuts and more sweets, which may explain the difference in the overall score.

Overall, the daily intake of fruits and vegetables was low in our study population. Only about 30% of young adults consumed a second serving of fruit and vegetables more than once a day. Previous studies conducted on similar young populations showed even lower consumption of fruits and vegetables. A survey conducted by McLean-Meyinsse et al. [15] on 305 college students reported that only 13% of students consumed fruits and vegetables at least two times per day, with 50% of the students consuming no fruits and 52% consuming no vegetables daily. Another recent study conducted on university students reported that two-thirds of students are not eating fruits and vegetables at all daily [16]. Put together, these findings suggest that young adults eat much less than the recommended amounts of fruits and vegetables. Although the Cypriot young adults studied here are not reaching the target of five portions of mixed fruit and vegetables a day (five-a-day) as per the Department of Health and other health agencies recommendations [17], it is encouraging that we at least report a higher consumption of fruit and vegetables than in other similar, non-Mediterranean, populations.

Our results indicate that a high percentage (76.2%) of Cypriot students consume at least two dairy products daily, complying with current dietary recommendations for dairy products (2-3 servings daily) [18]. Larson et al. [19] studied the changes in calcium and dairy intake during the transition from middle adolescence to young adulthood in a five-year follow-up study. According to their results, daily mean total intakes of dairy products were reduced by approximately 0.5 servings in both genders between baseline and follow-up. Also mean daily calcium intakes of females and males decreased by an average 153 mg and 194 mg, respectively. Reports in the literature further suggest that consumption of dairy products by children and adolescents in many countries has waned in recent decades and declines further with age [20–22]. Our findings are therefore encouraging and support the hypothesis that at least some aspects of the Mediterranean diet are still pursued in Mediterranean countries such as Cyprus, including dairy and pulses, which ~50% of study participants consume more than once per week. Furthermore the fact that our young population consumes dairy products may have even more clinical implications as yoghurt consumption may be beneficial for prevention of diabetes [23].

About a third of participating college students (30.6%) consumes sweets and candy several times a day. The high intakes of sweets and candy reported here are in line with similar findings from other studies in young populations [24, 25].

Another finding from our study was that a fourth of study participants (25.9%) visit a fast-food restaurant more than once a week. Avram and Oravitan [16] report very similar frequencies (26%) among 435 students from Timisoara University in New Zealand, while Niemeier et al. [26] in a prospective study of 9919 adolescents concluded that fast-food consumption and breakfast skipping increased during transition to adulthood, and both dietary behaviors were associated with increased weight gain from adolescence to adulthood.

Our study sample demonstrated a lower than recommended consumption of fruit and vegetables but a recommended daily consumption of dairy product. This is in accordance with—and perhaps explained by—very recently published data in a large sample of college students in Canada [27], showing that students believed they needed fewer vegetables and fruit and more milk than recommended quantities. Their findings further highlight the need for simpler age- and sex-specific recommendations and targeted campaigns.

While we report on the dietary habits and adherence to the Mediterranean diet of Cypriot young adults, it is also important to put our results in context with relevant findings from children in Cyprus, as reported from the CYKIDS study. The CYKIDS study [12] was a similar study conducted among school children (aged 9–13) in Cyprus during the school year 2004-2005, with a representative sample of school-aged children. Our study was conducted among young adults (aged 18–25) in 2014, making it very likely that the study sample described here comes from at least the same generation, after a time-span of 10 years. Although the sample of the CYKIDS study was representative of the total school-aged children's population, and our study was based on convenience sampling and therefore may not be representative of the total population of college students, we feel that comparisons are perhaps justified with regard to possible changes in dietary habits, prevalence of overweight and obesity, and adherence to the Mediterranean diet during the transition from childhood to young adulthood, especially as adherence to the Mediterranean diet was assessed using the same tool, the KIDMED index.

At childhood only 6.7% of the participants in the CYKIDS study were classified as high adherers to the Mediterranean diet, whereas 37% had a poor KIDMED index [28]. We report a lower prevalence of low adherence in adulthood (21.8%) and a much higher prevalence of high adherence to the Mediterranean diet (26.0% versus 6.7%) compared to the CYKIDS population [28]. Other differences between reports in the CYKIDS study and ours include a decrease in having breakfast and in the consumption of fruit and vegetables. Although no direct inferences can be made, given that our population is not necessarily representative of the Cypriot young adult population, it would be reasonable to assume that young college students may indeed skip breakfast more often now than as kids or eat less fruit and vegetables. Importantly though, we also show a relatively high daily consumption of dairy products in this young adult population as was also shown for the CYKIDS population. This finding may have further implications, as it may represent one food group that maintains a high consumption pattern through the transition from childhood to adulthood in Mediterranean populations. A recent report has highlighted the role of low-fat fermented dairy intake, and especially yogurt which had a high reported consumption in our population, with a reduction in the risk of developing type 2 diabetes, further implicating specific food groups in public health interventions [23].

Our study sample demonstrated a moderate coffee consumption (2-3 times per day) overall, with no big differences in consumption between genders (0.07), with more women though being in the lowest category (few times/year/never). Others have also reported a frequent consumption of coffee among young people with some gender differences [29, 30]. A review regarding caffeine consumption concluded that, for the healthy adult population, moderate daily caffeine intake at a dose level up to 400 mg/day is not associated with adverse effects [31]. A standard 8 oz (240 mL) cup of coffee is thought to have an average of 100 mg of caffeine [32]. However coffee consumption may affect diet quality in female college student. The average intakes of dietary fiber, vitamin A, beta-carotene, and folate in the noncoffee group have been reported to be significantly higher than those in the light coffee (<250 mL) and moderate coffee (≥250 mL) groups. Also the noncoffee group consumed a significantly higher amount of vegetables compared to the light coffee group [33].

With regard to alcohol consumption, we show that our study participants are occasional drinkers, in line with other studies [34, 35]. Reasons for drinking in youth include helping with their shyness, escaping their inhibitions, or as a way of being accepted by their peers. The main occasions of alcohol consumption are reported to be participation in social events and going out with friends [34]. Moreover a recent article indicated that students of permissive parents drank more beer and this was associated with more alcohol related problems. In agreement with our results, this study suggested that young women drank significantly less beer than young men [36].

Certain limitations of the study should be taken into account, especially its cross-sectional design, which limits any causal conclusions. In addition, study participants came from a convenience sampling from two university campuses and are not necessarily representative of the Cypriot college population. Although efforts were taken to ensure participation from both a public and a private university (thus trying to include students from all socioeconomic ranges), it is possible that our sample does not capture all the variability of the Cypriot college population. Data were based on self-reports, with some questionnaires not further validated, and therefore we cannot rule out the possibility of misreporting; however such methods are commonly used with similar studies and for the assessment of the main outcome (adherence to Mediterranean diet), a previously validated and widely used index was used.

## 5. Conclusion

To the best of our knowledge, this is the first study reporting on the dietary habits and adherence to the Mediterranean diet of Cypriot young adults and our results support the proposed shift from traditional healthy diets to more unhealthy eating patterns in Mediterranean countries with at least one-fifth of young adults having adopted poor dietary habits. Specifically, study participants consumed low quantities of fruits and vegetables, visit fast-food restaurants often, and consume sweets and candy several times a day. On the other hand, the majority of young adults in Cyprus still consume at least two dairy products daily and use olive oil at home, while also eating pulses more than once per week. Based on these findings, tailored-made public health strategies targeting the young adult population would be warranted, focusing on interventions to increase fruit and vegetable consumption and maintain dairy consumption, thus supporting adherence to the pattern of Mediterranean diet and reducing future burden of chronic diseases.

## Figures and Tables

**Table 1:** Baseline characteristics of study participants.

Variables	All 193	Males 87 (45.1%)	Females 106 (54.9%)	*p* value for difference between sexes
Age (yr)	20.56 ± 1.85	21.20 ± 1.59	20.05 ± 1.89	<0.001
Height (cm)	169.06 ± 9.53	176.66 ± 6.25	162.82 ± 6.86	<0.001
Weight (Kg)	67.09 ± 14.90	78.18 ± 12.37	57.98 ± 9.75	<0.001
BMI (Kg/m^2^)	23.31 ± 3.98	25.05 ± 3.68	21.89 ± 3.65	<0.001
BMI categories				
≤18.5 (Kg/m^2^)	6.2%	2.3%	9.4%	
18.5–25 (Kg/m^2^)	64.8%	51.7%	75.5%	
25–30 (Kg/m^2^)	24.9%	41.4%	11.3%	<0.001^*∗*^
>30 (Kg/m^2^)	4.1%	4.6%	3.8%	
Type of education				
Public university	94 (48.7%)	44 (50.6%)	50 (47.2%)	
Private university	99 (51.3%)	43 (49.4%)	56 (52.8%)	0.638
Currently employed				
Yes	47 (24.6)	27 (31.4%)	20 (19%)	
No	144 (75.4%)	59 (68.6%)	85 (81%)	0.049
Region of main residence				0.121^*∗*^
Nicosia	34 (17.6%)	20 (23%)	14 (13.2%)	
Larnaca	75 (38.9%)	34 (39.1%)	41 (38.7%)	
Limassol	65 (33.7%)	22 (25.3%)	43 (40.6%)	
Paphos	4 (2.1%)	2 (2.3%)	2 (1.9%)	
Famagusta	15 (7.8%)	9 (10.3%)	6 (5.7%)	
Currently exercising	107 (55.4%)	60 (69%)	47 (44.3%)	0.001
Currently smoking	46 (24%)	27 (31%)	19 (18.1%)	0.037
Protein supplements	24 (12.4%)	19 (21.8%)	5 (4.7%)	<0.001

Data are presented as mean ± SD and categorical variables as percentages in parentheses.

^*∗*^ *p* value for trend.

**Table 2:** Association between dietary/personal habits and mean BMI in all study participants.

Question asked	BMI (mean ± SD)	*p* ^*∗*^
*Do you have a fruit or fruit juice daily?*		
Yes (73.1%)	23.65 ± 3.94	
No (26.9%)	22.40 ± 3.98	0.054
*Do you have a second serving of fruit daily?*		
Yes (31.6%)	23.76 ± 3.07	
No (68.4%)	23.10 ± 4.33	0.292
*Do you eat fresh or cooked vegetables daily?*		
Yes (56.5%)	23.33 ± 3.70	
No (43.5%)	23.30 ± 4.34	0.961
*Do you eat fresh or cooked vegetables > 1/day?*		
Yes (29.5%)	24.14 ± 4.70	
No (70.5%)	22.97 ± 3.60	0.061
*Do you skip breakfast?*		
Yes (36.8%)	23.13 ± 4.40	
No (63.2%)	23.42 ± 3.73	0.626
*Doyou eat > 1/week at a fast-food restaurant?*		
Yes (25.9%)	23.85 ± 4.70	
No (74.1%)	23.13 ± 3.70	0.269
*Do you currently smoke? *		
Yes (24%)	22.69 ± 3.41	
No (76%)	23.53 ± 4.14	0.215
*Do you currently exercise? *		
Yes (55.4%)	23.30 ± 3.34	
No (44.6%)	23.32 ± 4.67	0.974
*Are you currently on a diet?*		
Yes (14.5%)	25.54 ± 5.18	
No (85.5%)	22.94 ± 3.62	0.001
*Do you currently take protein supplements?*		
Yes (12.4%)	23.74 ± 3.05	
No (87.6%)	23.25 ± 4.10	0.578

^*∗*^ *p* for difference in mean BMI in those who answered “Yes” versus those who answered “No” in the above questions regarding dietary/personal habits.

**Table 3:** KIDMED index questions in all and men and women separately. Positive answers are given as percentages (%).

KIDMED index questions	All (%)	Males (%)	Females (%)	*p* ^*∗*^
Fruit or fruit juice daily	73.1	77	69.8	0.26
Second serving of fruit daily	31.6	40.2	24.5	0.02
Fresh or cooked vegetables daily	56.5	55.2	57.5	0.74
Fresh or cooked vegetables > 1/day	29.5	32.2	27.4	0.47
Regular fish consumption (at least 2-3/week)	28	33.3	23.6	0.13
>1/week fast-food (hamburger) restaurant	25.9	31	21.7	0.14
Pulses > 1/week	47.7	50.6	45.3	0.46
Pasta or rice almost daily (≥5/week)	69.9	67.8	71.7	0.56
Cereal or cereal product for breakfast	73.1	70.1	75.5	0.40
Regular nut consumption (at least 2-3/week)	31.1	47.1	17.9	<0.001
Use of olive oil at home	87.6	87.4	87.7	0.94
No breakfast	36.8	36.8	36.8	0.99
Dairy product for breakfast	88.1	88.5	87.7	0.87
Commercially baked goods or pastries for breakfast	30.6	34.5	27.4	0.29
Two yoghurts and/or 40 gr cheese daily	76.2	72.4	79.2	0.27
Sweet and candy several times a day	30.6	24.1	35.8	0.04

^*∗*^ *p* value for comparison between sexes from chi-square test.

**Table 4:** Coffee, tea, and alcohol consumption in all and men and women separately.

	All (%)	Men (%)	Women (%)	*p* ^*∗*^
*Coffee consumption*				
4–6 times/day	8.4	11.5	5.8	
2-3 times/day	32.5	32.2	32.7	
1 time/day	23	25.3	21.2	
1–4 times/week	15.2	16.1	14.4	
1–3 times/month	7.3	9.2	5.8	
Few times/year or never	13.6	5.7	20.2	0.067
*Tea consumption*				
4–6 times/day	2.6	3.4	1.9	
2-3 times/day	7.9	8	7.7	
1 time/day	17.3	14.9	19.2	
1–4 times/week	23	29.9	17.3	
1–3 times/month	20.9	20.7	21.2	
Few times/year or never	28.3	23	32.7	0.329
*Wine consumption*				
4–6 times/day	1	1.1	1	
2-3 times/day	0.5	0	1	
1 time/day	5.8	5.7	5.8	
1–4 times/week	27.2	25.3	28.8	
1–3 times/month	26.7	35.6	19.2	
Few times/year or never	38.7	32.2	44.2	0.178
*Beer consumption*				
4–6 times/day	2.1	4.6	0	
2-3 times/day	0.5	1.1	0	
1 time/day	6.3	11.5	1.9	
1–4 times/week	26.2	39.1	15.4	
1–3 times/month	27.7	24.1	30.8	
Few times/year or never	37.2	19.5	51.9	<0.001
*Whiskey-vodka consumption*				
4–6 times/day	1	2.3	0	
2-3 times/day	1	2.3	0	
1 time/day	2.6	4.6	1	
1–4 times/week	19.9	25.3	15.4	
1–3 times/month	25.1	27.6	23.1	
Few times/year or never	50.3	37.9	60.6	0.012

^*∗*^ *p* value for comparison between sexes from chi-square test.
